# Pityriasis lichenoides et varioliformis acuta following anti-tetanus and diphtheria adult vaccine^☆^^☆☆^

**DOI:** 10.1016/j.abd.2019.06.009

**Published:** 2020-02-19

**Authors:** Maira Renata Merlotto, Natália Parente Bicudo, Mariangela Esther Alencar Marques, Silvio Alencar Marques

**Affiliations:** a Department of Dermatology and Radiotherapy, Faculdade de Medicina de Botucatu, Universidade Estadual Paulista, Botucatu, SP, Brazil; b Department of Pathology, Faculdade de Medicina de Botucatu, Universidade Estadual Paulista, Botucatu, SP, Brazil

Dear Editor,

Pityriasis lichenoides (PL) is a rare cutaneous inflammatory disease of unknown etiology consisting of three clinical forms: pityriasis lichenoides et varioliformis acuta (PLEVA), pityriasis lichenoides chronica and the severe febrile Mucha-Habermann's disease.^1^ PL affects mainly children and young adults. There is a relationship with response to extrinsic antigens, such as medications, infections, radiocontrast and vaccines. We present a very rare report of PLEVA associated with double-dose adult vaccine.

A 26-year-old male patient, previously healthy, complained of malaise, arthralgia, exanthema and 38 °C fever appearing two days after reinforcement with anti-tetanus and diphtheria adult vaccine (double-dose adult vaccine). Five days later, a dermatological examination revealed a polymorphous rash with generalized exanthema, associated with erythematous papules with adherent necrotic crusts and multiple hemorrhagic vesicles (Figure 1, Figure 2). The remainder of the physical examination was normal. Despite the patient's past medical history of varicella the diagnostic hypotheses were of hemorrhagic varicella and PLEVA. Serologies for HIV, HBV and HCV were negative. Tzanck test did not show multinucleated giant cells.

**Figure 1:**
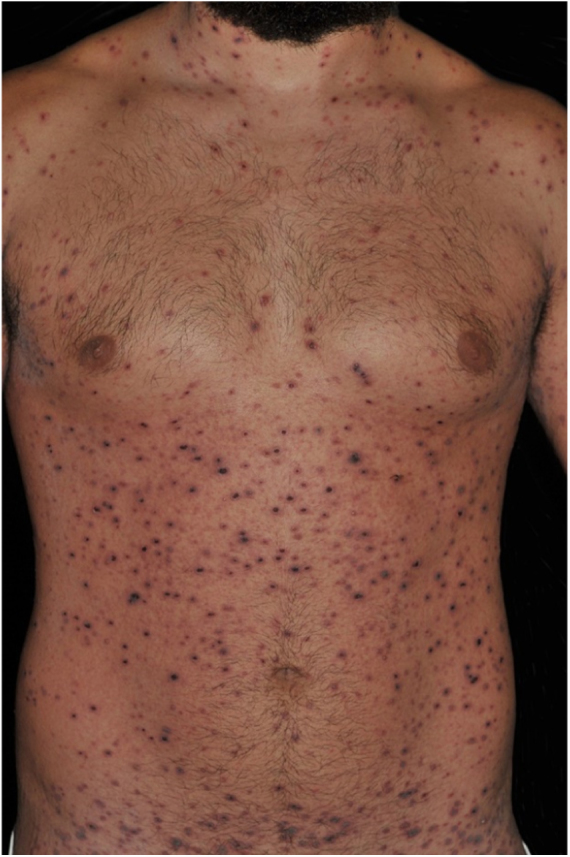
PLEVA: multiple hemorrhagic vesicles and papules with hematic crusts. Pre-treatment.

**Figure 2:**
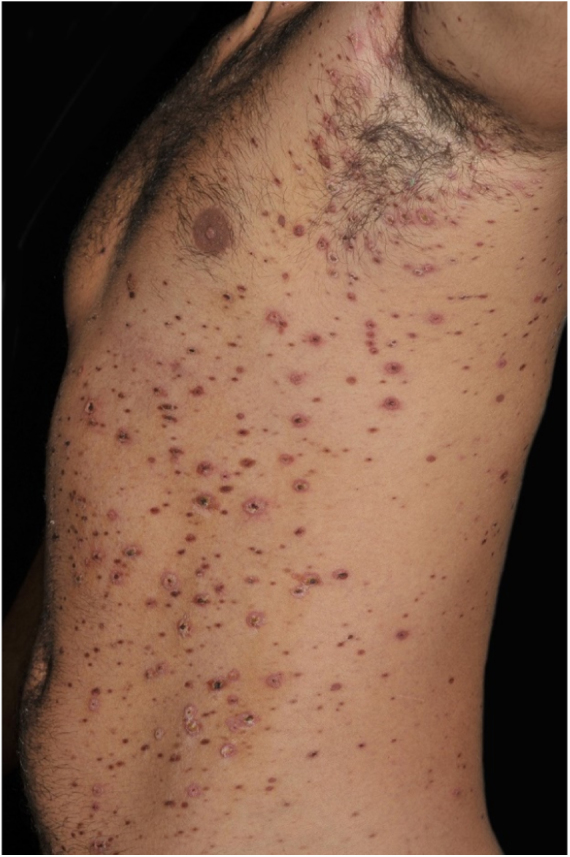
PLEVA: papulonecrotic lesions and papules with necrotic crusts. Treatment with metotrexate and systemic corticosteroids.

The histopathological examination revealed necrosis of the epidermis, suggestive signals of lymphocytic vasculitis and extravasation of red blood cells, supporting the clinical diagnosis of PLEVA (Fig. 3). With the diagnosis of severe PLEVA, we started treatment with prednisone 1 mg/kg/day plus potassium permanganate wet dressing. As no improvement was obtained, methotrexate (15mg/week) was added a week later. A considerable and fast improvement was observed with this regimen. In the follow-up, corticosteroid was tapered and, two months later, methotrexate was discontinued. Achieving clinical cure, although with multiple residual varioliform scars.

**Figure 3:**
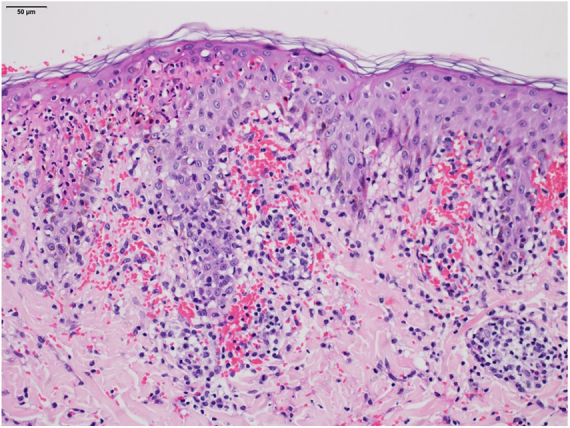
PLEVA: histopathological examination reveals necrosis of the epidermis, suggestive signals of lymphocytic vasculitis and large number of extravasated red blood cells (Hematoxylin & eosin, x200).

PLEVA is characterized by a polymorphous eruption of erythematous macules that rapidly evolve into 2–3 mm papules, vesicles, and vesicopustular or hemorrhagic lesions, which undergo necrosis with overlapping crusts. It can result in varioliform scars. Symptoms include burning and itching. The Mucha–Habermann subtype is an intense, varicella-like, ulceronecrotic cutaneous disease associated with systemic repercussions, fever and impairment of clinical condition. Our case would be situated between the two acute subtypes of the disease.

PL is caused either by an inflammatory reaction triggered by extrinsic factors or has lymphoproliferative origin, as an inflammatory response secondary to a T-cell dyscrasia; or, yet, immune complex mediated hypersensitivity vasculitis.^1^ In this case, the first hypothesis seems to be more plausible. In an extensive literature review, we found only three reports associating PLEVA with triple viral vaccine and vaccine against influenza.^2^, ^3^, ^4^ To the best of our knowledge, our patient is the first to show the association of PLEVA and double-dose adult vaccine. The double-dose adult vaccine is composed of a combination of diphtheria and tetanus toxoid, aluminum hydroxide or phosphate as an adjuvant, and thimerosal as a preservative, administered intramuscularly deep in the deltoid.^5^ Treatments for PL are based on uncontrolled case series or case reports. First-line therapy includes topical corticosteroids, tetracycline, erythromycin, and various types of phototherapy. In severe and/or fulminant cases, the use of systemic corticosteroids, methotrexate or ciclosporin may be necessary.^1^ We emphasize the favorable response to the established therapy in this case.

## Financial support

None declared.

## Authors’ contributions

Maira Renata Merlotto: Conception and planning of the study; elaboration and writing of the manuscript; review of the literature; critical review of the manuscript.

Natália Parente Bicudo: Approval of the final version of the manuscript; obtaining, analysis, and interpretation of the data; review of the literature; critical review of the manuscript.

Mariangela Esther Alencar Marques: Approval of the final version of the manuscript; obtaining, analysis, and interpretation of the data; critical review of the manuscript.

Silvio Alencar Marques: Approval of the final version of the manuscript; elaboration and writing of the manuscript; critical review of the manuscript.

## Conflicts of interest

None declared.

## References

[1] Bowers S., Warshaw E.M. (2006). Pityriasis lichenoides and its subtypes. J Am Acad Dermatol.

[2] Castro B.A., Pereira J.M., Meyer R.L., Trindade F.M., Pedrosa M.S., Piancastelli A.C. (2015). Pityriasis lichenoides et varioliformis acuta after influenza vaccine. An Bras Dermatol.

[3] Gunatheesan S., Ferguson J., Mossa Y. (2012). Pityriasis lichenoides et varioliformis acuta: a rare association with the measles, mumps and rubella vaccine. Australas J Dermatol.

[4] Gil Bistes D., Kluger N., Bessis D., Guillot B., Raison-Peyron N. (2012). Pityriasis lichenoides chronic after measles-mumps-rubella vaccination. J Dermatol.

[5] Weinberger B. (2017). Adult vaccination against tetanus and diphtheria: the European perspective. Clin Exp Immunol.

